# Statistical Analysis of Toxicological Data of Victims of Traffic Accidents in Galicia (Spain)

**DOI:** 10.1007/s11121-023-01502-8

**Published:** 2023-02-09

**Authors:** Iván Alvarez-Freire, Olga López-Guarnido, Pamela Cabarcos-Fernández, Manuel Couce-Sánchez, Ana María Bermejo-Barrera, María Jesús Tabernero-Duque

**Affiliations:** 1 Forensic Toxicology Service. Forensic Sciences Institute, Universidad de Santiago de Compostela. C/San Francisco S/N, 15782 Santiago de Compostela, Spain; 2 Department of Legal Medicine and Toxicology, Medical School, University of Granada, Avenida de La Investigación nº 11, 18071 Granada, Spain

**Keywords:** Traffic accidents, Alcohol, Drugs, Statistics, Toxicological data

## Abstract

Driving under the influence of alcohol or drugs is a very common behavior in our environment and a serious problem for public health. On the one hand, in 2016, 400,000 people died in the world in traffic accidents in which ethanol was involved. On the other hand, traffic accidents in which the use of drugs of abuse other than ethyl alcohol accounted for more than 160,000 deaths worldwide in 2017. The objective of this work is to carry out a review of the 710 cases of people who died in traffic accidents received at the forensic toxicology service of the Institute of Forensic Sciences of the University of Santiago de Compostela (Galicia-Spain) over a period of 10 years (2009–2019). We performed an observational study of period prevalence, in which the following data were collected: age, sex, year, and analytical results in plasma, in the case of being positive. The data collected was subjected to statistical treatment. Of the 710 cases analyzed, 123 correspond to pedestrians and 587 to occupants of vehicles or motorcycles. A total of 77.6% of the deceased were men. At least one psychotropic substance was found in the blood of almost 40% of the victims. The most frequently found substance was ethyl alcohol, which appeared in 231 cases, more frequently in males. The second place is occupied by benzodiazepines, which appeared in 43 cases, followed by cocaine, which was detected in 25 cases. Polydrug use was found in only 44 cases, with the association of ethanol and cocaine being the most commonly found, followed by that of ethanol and benzodiazepines. Only in 5 of the cases analyzed there were 3 or more substances present. With the data obtained in this study, it is shown that in traffic accidents, the finding of different toxic or medicinal substances is frequent. Ethyl alcohol continues to be very present in road accidents (most detected substance), with the great impact that this implies. Secondly, the presence of benzodiazepines stands out, and cocaine is the third most detected toxic in this study. These results allow to obtain a profile of the substances most frequently involved in traffic accidents. Despite the surveillance, control, and information campaigns that the Spanish Government regularly carries out, the results are far from satisfactory.

## Introduction

Driving under the influence (DUI) of alcohol or drugs is a serious threat to public safety. Every year, 1.3 million people die because of road traffic crashes (WHO, 2021). Although many of these accidents are due to improper speeding, it is well known that driving under the influence of alcohol and/or any psychoactive substance or drug increases the risk of a traffic accident that results in death or serious injuries.

Alcohol consumption is one of the leading causes of death and disability in the world. Three million people died in 2016 from alcohol, of which almost 400,000 were due to traffic accidents related to alcohol consumption. Half of these deaths from accidents and alcohol were non-driver users. In Europe, alcohol is related to around 20 to 25% of all road deaths (WHO, 2021; European Transport Safety Council, 2022).

For its part, consumption of drugs of abuse other than alcohol accounted for more than 160,000 deaths throughout the world in 2017, and it is estimated that illegal drugs caused around 40,000 deaths per traffic accident during 2013. Drug use in Europe now encompasses a wider range of substances than in the past, being the prevalence of cannabis use approximately five times higher than that of other substances. The prevalence of drugs among injured drivers or deaths in traffic accidents in Western countries is not uncommon and is estimated to range from 14 to 17% (European Monitoring Centre for Drugs and Drug Addiction, 2019; Achermann Stürmer, 2016).

Several studies have shown that alcohol and illicit drugs affect brain functions essential to a difficult task like driving and increase the chances of being in a car accident (Ferrari et al., 2018; Steuer Andrea et al., 2016; Ji Kwon & Han, 2019). However, although the effect of alcohol on driving skills has been extensively studied, the literature on illegal drugs is generally scarce, and there are still debates about the effects of these substances on driving skills (Ferrari et al., 2018).

Toxicological analysis plays an essential role in the investigation of practically all forensic cases, being an integral component of the investigation of traffic accidents. Findings found during toxicology tests can clarify the cause of the crash. Statistical studies can add useful information to avoid the serious problem of road accidents by providing data information on the most usually consumed substances involved in these deaths.

In the Forensic Toxicology Laboratory of the Forensic Sciences Institute of the University of Santiago de Compostela, samples are sent from coroners to investigate the deceased in Galicia, a region in the northwest of Spain, with an estimated population of 2.7 million inhabitants. Except for the reports from the Spanish government to our knowledge, there are no studies of traffic accidents from the toxicological point of view in Galicia.

The objective of this work is to carry out a review of the cases of people who died in traffic accidents (including pedestrians) received in our service in a period of 10 years (2009–2019).

To carry out this work, no new toxicological analyses have been carried out. Only strictly necessary data have been reviewed and collected. A total of 710 cases were reviewed, all of which were analyzed in our laboratory. For this review, the following data were collected: age, sex, year, and analytical results in plasma, if positive. The collected data were subjected to statistical treatment with the SPSS v26® program.

## Material and Methods

### Study Design and Inclusion Criteria

This is an observational study of period prevalence. For this study, the toxicological results in the blood of 710 cases of death due to a traffic accident were included. These were all the cases received in our laboratory during the studied period. The samples were collected in the autopsies carried out at the IMELGA (Institute of Legal Medicine of Galicia) and analyzed in the Forensic Toxicology Laboratory of the University of Santiago de Compostela (Spain) from 2009 to 2019. The collection of biological samples from autopsies is mandatory, as required by the judicial authority. The analyses are requested from our laboratory by the Ministry of Justice for the purpose of forensic expertise, and it is the forensic doctors’ responsibility to collect, send, and keep the samples.

### Toxicological Analyses

Blood was collected into plastic tubes (BD Vacutainer®, -gray or lavender cap- provided with sodium fluoride as a preservative and ethylendiamine tetraacetic acid (EDTA) or potassium oxalate as an anticoagulant. Samples were sent to the laboratory in refrigerated boxes, and there were stored at 4 °C until the analysis was completed. Once the analysis was finished the samples were kept at −20 °C.

For traffic accident deaths, a systematic toxicological analysis of ethanol, drugs of abuse, and therapeutic drugs were performed. The concentration of ethanol in blood was measured by gas chromatography-flame ionization detection (Agilent 7820A, Agilent Technologies). Screening procedures in urine include immunoassays (Cobas Integra 400, Roche) and gas chromatography coupled to mass spectrometry (GC/MS 7890B/5977B Agilent®) analysis in full scan mode. The cut-off levels for the immunoassay screening were cocaine (300 ng/mL), opiates (300 ng/mL), cannabinoids (50 ng/mL), amphetamines (1000 ng/mL), methadone (300 ng/mL), and benzodiazepines (100 ng/mL). All positive results after screening in urine were confirmed in blood by chromatographic analysis using the GC/MS in SIM mode, and liquid chromatography coupled to a diode array detector (HPLC–DAD, Waters®) through a quantitative analysis. Drugs were identified using commercial libraries. In this work, the result of blood analysis that detects the presence of any drug of abuse or psychotropic drug is considered positive, after confirmation of the preliminary screening, without considering the amount detected. The analytical methods used are the standard methods used in the laboratory at the time of performing the analyses.

### Studied Variables

The qualitative variables were benzodiazepines consumption (no/yes), cocaine consumption (no/yes), cannabis consumption (no/yes), ethanol consumption (no/yes), pharmaceutical drugs consumption (no/yes), toxics consumption (no/yes), polydrug use (no/yes), type of accident (vehicle occupant/pedestrian), and sex (female/male). The toxic consumption variable was given a “yes” value if any toxic substance was detected (illegal and pharmaceutical drugs including ethanol). The polydrug use variable was given a “yes” value if two or more toxic substances were detected (illegal and pharmaceutical drugs and ethanol). The quantitative variables were age (years) and ethanol concentration (g/l). The age variable was categorized into different levels.

### Statistical Analysis

The results were analyzed using the IBM® SPSS® software version 26.0. A database was created with the collected information. A descriptive analysis of the characteristics of the study population was performed using measures of central tendency (mean, median, and standard deviation) for continuous variables (age and ethanol level) and proportions for categorical variables (gender, ethanol consumption, etc.). We used the chi-square test or Fisher’s correction to compare the consumption of the substances under study with gender and type of accident (we used Fisher’s correction when the minimum expected count was lower than 5 and the chi-square test otherwise). Since quantitative variables (age and ethanol concentration) did not follow a normal distribution (Kolmogorov–Smirnov test), we used Kruskal–Wallis or Mann–Whitney U tests for bivariate comparisons. If the Kruskal–Wallis test was significant, and with the aim of determining differences between specific groups, we performed pair-wise comparisons using Dunn’s procedure with a Bonferroni correction. The level of statistical significance was established for a value of *p* < 0.05.

## Results

### Sample Characteristics: Type of Accident, Gender, and Age of the Victims

A total of 22.4% (*N* = 159) of the 710 cases studied are women, and 77.6% are men (*N* = 551). The majority (82.7%, *N* = 587) of fatalities in our sample are vehicle occupants compared to only 17.3% (*N* = 123) that are pedestrians. The proportion of male vehicle occupants (66.9%; *N* = 475) is significantly higher than the proportion of females (15.8%, *N* = 112, *p* = 0.000).

Table 1 shows that the age of 9 individuals is not available, and of the remaining 701 the mean was 50.86 years (± SD = 20.47), with a minimum value of 2 and a maximum of 101. The median age for women (Mdn = 60) is significantly higher than for men (Mdn = 47, *p* = 0.000). The age of the vehicle occupants (Mdn = 45) was significantly lower than the age of the pedestrians (Mdn = 71, *p* = 0.000).

**Table 1:** Age distribution with the main variables

		**N**	**Mean**	** ± SD**	**Median**	**Mínimum**	**Maximum**	**IQR**	*p*-value*
	Total	701	50.86	20.47	50	2	101	32	
Sex	Women	156	57.12	21.10	60	14	90	35	0.000
	Men	545	49.07	19.95	47	2	101	32	
Type of accident	Vehicle occupants	578	47.4	19.35	45	14	101	31	0.000
	Pedestrians	123	67.12	17.60	71	2	95	24	
Benzodiazepines	No	658	50.53	20.54	49.5	2	101	33	0.098
	Yes	43	56	18.79	51	20	90	34	
Cocaine	No	677	51.47	20.48	50	2	101	34	0.000
	Yes	24	33.63	10.4	34	19	54	18	
Cannabis	No	684	51.36	20.42	50	2	101	33	0.000
	Yes	17	30.82	10.11	27	19	48	18	
Alcohol	No	472	53.51	21.23	53	2	101	35	0.000
	Yes	229	45.41	17.63	44	17	90	30	
Pharmaceutical Drugs	No	648	50.44	20.59	49	2	101	33	0.053
	Yes	53	56.09	18.2	52	20	90	33	
Illegal Drugs	No	662	51.89	20.46	51	2	101	33	0.000
	Yes	39	33.41	10.2	34	19	54	18	
Toxic consumption^1^	No	424	53.58	21.30	54	2	101	35	0.000
	Yes	277	46.7	18.40	46	17	90	29	
Polydrug Use	No	658	51.59	20.62	51	2	101	34	0.000
	Yes	43	39.70	14.1	40	19	83	18	

*N *number of individuals, *IQR *interquartile range, *SD *standard deviation

^1^considering ethanol

*U de Mann–Whitney

### Toxicological Results

Regarding the analytical results, 341 tests were positive in a total of 279 individuals (39.3%) with at least one positive result. From all the substances analyzed, the most consumed was alcohol (*N* = 231, 67.7%), followed by benzodiazepines (*N* = 43, 12.6%), cocaine (*N* = 25, 7.3%), cannabis (*N* = 17, 5%), methadone (*N* = 7, 2.1%), antidepressants (*N* = 6, 1.6%), and opiates (*N* = 5, 1.5%). Other substances detected in the samples analyzed were anticonvulsants (*N* = 3, 1%), analgesics (*N* = 2, 0.6%), barbiturates (*N* = 1, 0.3%), and amphetamines (*N* = 1, 0.3%).

It was found that the consumption of at least one toxic substance was significantly related to sex, type of accident (Table 2), and age (Table 1). In relation to gender (Table 2), the proportion of female consumers (*N* = 32, 20.1%) was significantly lower (*p* = 0.000) than the proportion of male consumers (*N* = 247, 44.8%). In relation to the type of accident, the proportion of vehicle occupants that consumed any toxic substance (41.1%, *N* = 241) was significantly higher than in pedestrian consumers (30.9%, *N* = 38; *p* = 0.042). Regarding age (Table 1), it is significantly lower (Mdn = 46) in the group of consumers of some toxic than in the group of non-users (Mdn = 54; *p* = 0.000).

**Table 2:** Substances found vs gender and type of accident

		**N (%)**	**Gender**		***p-value***	**Type of accident**		***p-value***
			**Women**	**Men**		**Vehicle occupants**	**Pedestrians**	
			**N (%)**	**N (%)**		**N (%)**	**N (%)**	
**Toxics consumption**^***1***^	No	431 (60.7%)	127 (79.9%)	304 (55.2%)	0.000^*2*^	346 (58.9%)	85 (69.1%)	0.042 ^*2*^
	Yes	279 (39.3%)	32 (20.1%)	247 (44.8%)		241 (41.1%)	38 (30.9%)	
**Pharmaceutical Drugs**	No	657 (92.5%)	143 (89.9%)	514 (93.3%)	0.171^*2*^	543 (92.5%)	114 (92.7%)	0.945^*2*^
	Yes	53 (7.5%)	16 (10.1%)	37 (6.7%)		44 (7.5%)	9 (7.3%)	
**Benzodiazepines**	No	667 (94%)	146 (91.8%)	521 (94.6%)	0.256^*2*^	552 (94.0%)	115 (93.5%)	0.0835^*2*^
	Yes	43 (6%)	13 (8.2%)	30 (5.4%)		35 (6.0%)	8 (6.5%)	
**Illegal Drugs**	No	670 (94.4%)	157 (98.7%)	513 (93.1%)	0.010^*2*^	547 (93.2%)	123 (100%)	0.005^*2*^
	Yes	40 (5.6%)	2 (1.3%)	38 (6.9%)		40 (6.8%)	0 (0%)	
**Cocaine**	No	685 (96.5%)	157 (98.7%)	528 (95.8%)	0.089^*2*^	562 (95.7%)	123 (100%)	0.013^*3*^
	Yes	25 (3.5%)	2 (1.3%)	23 (4.2%)		25 (4.3%)	0 (0%)	
**Cannabis**	No	693 (97.6%)	159 (100%)	534 (96.9%)	0.018^*3*^	570 (97.1%)	123 (100%)	0.055^*3*^
	Yes	17 (2.4%)	0 (0%)	17 (3.1%)		17 (2.9%)	0 (0%)	
**Polydrug Use **^**1**^	No	666(93.8%)	158 (99.4%)	508 (92.2%)	0.001^*2*^	545 (92.8%)	121 (98.4%)	0.021^*2*^
	Yes	44 (6.2%)	1 (0.6%)	43 (7.8%)		42 (7.2%)	2 (1.6%)	

*N *number of individuals

% percentage referring to the compared groups

^*1*^considering alcohol as toxic

^*2*^chi-square

^*3*^Fisher

#### Pharmaceutical Drugs

At least one pharmaceutical drug (benzodiazepines, barbiturates, antidepressants, anticonvulsants, analgesics) was detected in 53 individuals (7.5%). Two of them were consumers of two pharmaceutical drugs (specifically benzodiazepines and antidepressants). Medication use is not significantly related to gender (Table 2), type of accident (Table 2), or age (Table 1).

In this study population, benzodiazepines are the most consumed medication, and it was observed in 43 individuals. The most frequently found benzodiazepines were nordazepam, oxazepam, diazepam, and bromazepam. Midazolam was found in 5 cases, probably administered by the emergency services before death. They are also the second most consumed toxic after alcohol. In most benzodiazepine users (65.1%; *N* = 28), no other toxic substance was detected.

No statistically significant relationship was found between the consumption of benzodiazepines and type of accident and gender (Table 2) and age (Table 1).

#### Ethanol

Alcohol was the most detected substance, appearing in 32.5% of individuals (*N* = 231). In many of the cases (83.1%; *N* = 192), alcohol was detected alone.

The age of alcohol users (Mdn = 44) (Table 1) is significantly lower than the one of non-consumers (Mdn = 53; *p* = 0.000).

The association between alcohol and gender (Table 3) is significant (*p* = 0.000), finding that the proportion of female consumers (9.4% *N* = 15) is significantly lower than the proportion of male consumers (39.2%, *N* = 216). The association between alcohol and type of accident (Table 3) is not significant, but borders on significance (*p* = 0.058). Most of the alcohol consumers (86.6%) were vehicle occupants (*N* = 200).

**Table 3:** Relations between ethanol and the main variables (gender, type of accident, and drugs)

-		Ethanol	
		No	Yes
N		479	231
Gender	Women N (%)	144 (90.6)	15 (9.4%)
	Men N (%)	335(60.8%)	216 (39.2%)
p- value*		0	
Type of accident	Ocupante N (%)	387 (65.9%)	200 (34.1%)
	Atropellado N (%)	92 (74.8%)	31 (25.2%)
p- value*		0.058	
Cocaine	No N (%)	472 (68.9%)	213 (31.1%)
	Yes N (%)	7 (28%)	18 (72%)
p- value*		0	
Cannabis	No N (%)	474 (68.4%)	219 (31.6%)
	Yes N (%)	5 (29.4%)	12 (70.6%)
p- value*		0.001	
Benzodiazepines	No N (%)	447 (67%)	220 (33%)
	Yes N (%)	32 (74.4%)	11 (25.6%)
p- value*		0.401	
*Polydrug Use*	No N (%)	474 (71.2%)	192 (28.8%)
	Yes N (%)	5 (11.4%)	39 (88.6%)
p- value*		0	

*N *number of individuals

% percentage referring to the compared groups

^*^Chi-square

Regarding ethanol concentration (Table 4), the average value is 1.698 g/l with a minimum value of 0.04 g/l and a maximum of 5.650 g/l. By categorizing the concentration of ethanol at different levels, we found that only 6.5% (*N* = 46) of the ethanol-positive individuals had less than 0.5 g/l, 10% (*N* = 71) had a concentration between 0.5 and 1.7 g/l, 8.3% (*N* = 59) had a concentration between 1.701 and 2.5 g/l, 6.2% (*N* = 44) between 2.501 and 3.5 g/l, and 1.5% (*N* = 11) had an ethanol concentration greater than 3.5 g/l.

**Table 4:** Ethanol concentration (g/l)

		N	Mean	± SD	Median	Minimum	Maximum	IQR	*p*-value*
		231	1.698	1.128	1.700	0.040	5.650	1.85	
Sex	Women	15	1.079	0.7	0.84	0.2	2.35	1.16	0.031
	Men	216	1.741	1.14	1.775	0.04	5.65	1.89	
Type of accident	Vehicle occupant	200	1.687	1.118	1.69	0.04	5.65	1.795	0.625
	Pedestrians	31	1.763	1.206	1.75	0.085	3.78	2.43	
Benzodiazepines	No	220	1.701	1.135	1.69	0.04	5.65	1.865	0.932
	Yes	11	1.622	1.015	1.78	0.12	3.2	1.69	
Cocaine	No	213	1.681	1.129	1.67	0.04	5.65	1.875	0.486
	Yes	18	1.898	1.13	1.8	0.16	5.05	1.372	
Cannabis	No	209	1.714	1.145	1.68	0.04	5.65	1.9	0.413
	Yes	12	1.397	0.712	1.72	0.17	2.3	1.275	
Pharmaceutical drugs	No	209	1.707	1.134	1.7	0.04	5.65	1.85	0.667
	Yes	12	1.521	1.029	1.465	0.12	3.2	1.992	
Illegal drugs	No	203	1.701	1.138	1.67	0.04	5.65	1.87	0.878
	Yes	28	1.676	1.074	1.77	0.09	5.05	1.398	
Polydrug use	No	192	1.716	1.144	1.675	0.040	5.650	1.915	0.583
	Yes	39	1.606	1.053	1.053	0.090	5.050	1.42	

*N* number of individuals, *IQR* interquartile range, *SD* standard deviation

*U de Mann–Whitney

**Fig. 1:**
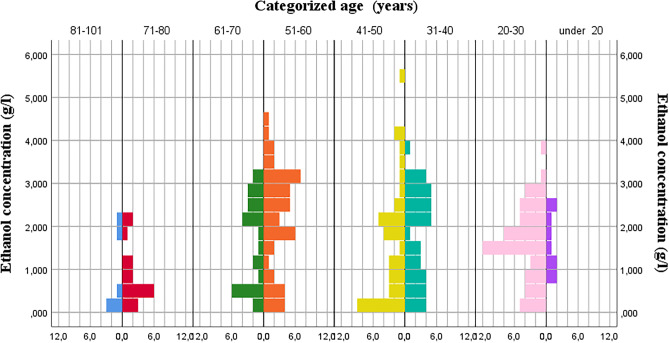
Frequency of ethanol concentration (g/l) by categorized age

The ethanol concentration (Table 4) in women (Mdn = 0.84) is significantly lower than in men (Mdn = 1.775; *p* = 0.031). Comparing the ethanol concentration and the rest of the variables studied (type of accident, benzodiazepines, cocaine, cannabis, pharmaceutical drugs, illegal drugs, and polydrug use), it was not statistically significant (Table 4). When studying the association between the ethanol concentration and the categorized age (Table 5), the results show that the distributions of ethanol concentration were significantly different between the groups (*p* = 0.001). To determine differences between age groups, a post-hoc analysis was performed (pair-wise comparisons using Dunn’s procedure with a Bonferroni correction), and it revealed statistically significant differences in ethanol concentration between the age group 71–80 years (Mdn = 0.575) and the age group 51–60 years (Mdn = 2.360) (*p* = 0.001), but not in other combinations (Fig. 1).

**Table 5:** Comparison of ethanol concentration (g/l) and categorized age

		**N**	**Mean**	** ± SD**	**Median**	**Minimum**	**Maximum**	**IQR**	*p*-value*
**Age (years)**	< 20	9	1.640	0.734	1.490	0.810	2.640	1.520	0.001
	20–30	52	1.598	0.847	1.595	0.040	3.700	1.128	
	31–40	39	1.782	1.069	2.120	0.160	3.900	1.990	
	41–50	37	1.655	1.960	1.470	0.090	5.650	2.045	
	51–60	45	2.196	1.169	2.360	0.160	4.500	1.875	
	61–70	25	1.629	1.044	1.800	0.085	3.270	2.035	
	71–80	16	0.856	0.676	0.575	0.150	2.290	0.905	
	81–101	6	0.808	0.816	0.335	0.230	2.030	1.515	

*N* number of individuals, *IQR* interquartile range, *SD* standard deviation

*Kruskal–Wallis

#### Illegal Drugs

It was found that 5.6% (*N* = 40) of individuals have used at least one illegal drug, and it was significantly related to gender, type of accident (Table 2), and age (Table 1). In relation to gender, the proportion of male drug users (*N* = 38, 6.9%) is significantly higher than the proportion of female drug users (*N* = 2, 1.3%) (*p* = 0.01). There is a significant association (*p* = 0.0050) between using or not using illegal drugs and the type of accident: in fact, we did not find any pedestrian that used any illegal drug. Age (Table 1) is significantly higher in the non-drug use group (Mdn = 51) than in the drug use group (Mdn = 34; *p* = 0.000).

##### Cocaine

Cocaine is the third most detected toxic in this study, being found in 25 individuals (3.5%). Cocaine use (Table 2) is related to the type of accident; in fact, there are no pedestrians positive in cocaine (*p* = 0.013). On the other hand, although the proportion of male consumers (4.2%, *N* = 23) is higher than female consumers (1.3%, *N* = 2), the differences are not statistically significant (*p* = 0.089). The group of non-consumers is significantly older (Mdn = 50) than the users (Mdn = 34, *p* = 0.000) (Table 1).

##### Cannabis

It is the fourth most frequently detected substance in our population (*N* = 17, 2.4%). Cannabis use (Table 2) is significantly related to gender (*p* = 0.018) and borders on significance with the type of accident (*p* = 0.055); in fact, there is no woman, nor any pedestrian in our sample who is a consumer of cannabis. Age (Table 1) is significantly higher in non-consumers of cannabis (Mdn = 50) than those who do (Mdn = 27; *p* = 0.000).

##### Associations of Ethanol with Other Substances

In relation to the consumption of ethanol and other toxic substances (Table 3), we found that there is a significant relationship between the consumption of cocaine and the consumption of cannabis, but not with benzodiazepines. Thus, among cocaine users, the proportion of alcohol users is significantly higher (*N* = 18, 72%) than the proportion of non-alcohol users (*N* = 7, 28%). The consumers of both alcohol and cocaine were all male and all occupants of the vehicle. Among cannabis users, the proportion of alcohol users is significantly higher (*N* = 12, 70.6%) than the proportion of non-alcohol users (*N* = 5, 29.4%).

##### Polydrug use

**Fig. 2:**
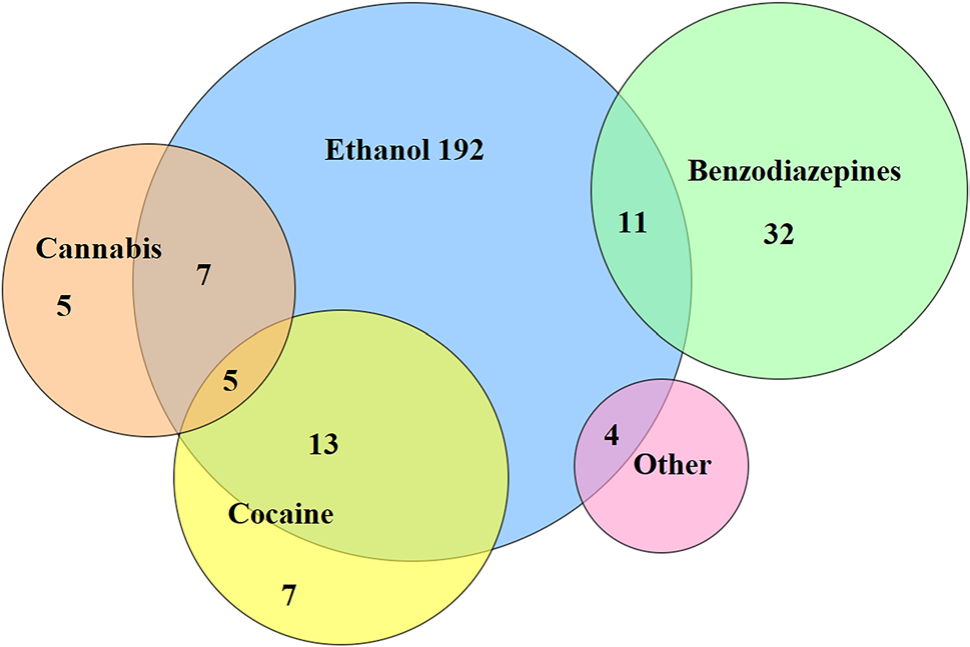
Venn diagram of ethanol-positive deceased. Classification of the results by type and combination of the main substances found

From the 279 individuals in the sample who tested positive for some toxic, the vast majority (84.2%, *N* = 235) tested positive for a single substance, and only 15.8% (*N* = 44) of the sample were polydrug users (individuals who consume 2 or more substances). Table 2 shows how polydrug use is related to sex (*p* = 0.001) and to the type of accident (*p* = 0.021). Among individuals who are polydrug users, the most consumed substance is alcohol, consumed 39 times. The most frequently found association is between alcohol and cocaine (*N* = 18) (Fig. 2).

## Discussion

During the 10-year period from 2009 to 2019, the total number of accidents with fatalities has been gradually decreasing, both in Spain and in Galicia, going from 2714 deaths in 2009 to 1755 in 2019 throughout Spain. This is due in part to the application of traffic laws, which have contributed significantly to improving road safety in Spain. The number of drunk driving checks by the traffic police (Guardia Civil) increased from 5.5 million in 2018 to 6.5 million in 2019. In 2007, Spain introduced the first checks on drug driving as it participated in the European project DRUID during the period 2008–2011. Currently, the Spanish authorities are focusing on speed limit changes in an effort to reduce road deaths. On May 11, 2021, the default speed limit was lowered from 50 to 30 km/h on single-carriageway urban roads and to 20 km/h in shared spaces (ETSC, 2022).

In this study, alcohol and other drugs are still intimately linked to traffic accidents. In fact, alcohol was the most detected drug, appearing in 231 cases out of 710.

In our study, the most detected substance is alcohol, which is consistent with other studies about toxic substances consumption in victims of mortal traffic accidents (Morland et al., 2011; Legrand et al., 2014; Biecheler et al., 2008; Holmgren et al., 2005; Elliott et al., 2009; del Rio et al., 2002; National Institute of Toxicology and Forensic Sciences, 2020). Regarding other similar studies in Spain, alcohol-positive results are even very similar, being 32.53% (*N* = 231) in our study and 29.5% (*N* = 216) in the National Institute of Toxicology Report (National Institute of Toxicology and Forensic Sciences, 2020).

We also found that men consume more alcohol than women, which is the same result as other studies that consider the gender differences in alcohol intake in deadly traffic accidents (Legrand et al., 2014; Holmgren et al., 2005; Elliott et al., 2009).

The high average blood alcohol concentration (BAC) found in our study (1.698 g/L) is worrying, which indicates that a large part of the deceased is heavy drinkers or alcoholics. In 192 cases, ethanol was found as the only substance consumed, and in 55 cases had a BAC greater than 2.50 g/L. The age group with the highest number of positives was 20–30.

In this study, the most detected illegal drug is cocaine (3,5%, *N* = 25), which is in concordance with other studies in Spain (Elliott et al., 2009; del Rio et al., 2002) although if studies carried out on live drivers in Spain are consulted, the most frequent drug found in cannabis (Domingo-Salvany et al., 2017; Herrera-Gómez et al., 2020). Nevertheless, other European studies carried out on traffic victims’ frequent illegal drug is different: in the study of Holmgren et al. (2005) and Legrand et al. (2014) they were amphetamines, and in the work of Elliott et al. (2009) and Morland et al. (2011), it was cannabis.

The most consumed prescription in our study were benzodiazepines (6,05%, *N* = 43), which is also the most frequent in the other studies performed in Spain (del Rio et al., 2002; National Institute of Toxicology and Forensic Sciences, 2020) and in most European studies (Morland et al., 2011; Legrand et al., 2014) while in Holmgren et al. (2005), benzodiazepines are the second most consumed medicine after antidepressants, albeit with a similar rate. In our study, benzodiazepines are also the second most consumed substance after alcohol, which is also the case in other published studies (Morland et al., 2011; Legrand et al., 2014; del Rio et al., 2002; National Institute of Toxicology and Forensic Sciences, 2020).

Polydrug use is a high risk for dangerous driving (Snenghi et al., 2018). In this study, there were 44 cases, 39 in which alcohol was present, and 5 in which it was not. Cocaine and ethanol (18 cases), cannabis and ethanol (12 cases), and ethanol and benzodiacepines (11 cases) were the combinations more frequently found. The most frequent combination is cocaine and alcohol. The concurrent use of cocaine and ethanol results in a biologically active molecule, cocaethylene (CE), which is nearly as psychoactive as COC but produces a more long-lasting high (Álvarez et al., 2007). Moreover, CE is even more toxic than COC, and its potency results in an increased risk of death due to overdose.

This study has been carried out only with data on fatalities in traffic accidents and does not include information on injured people. Although this may be a limitation (since data on all accident victims are not available), the results are significant enough to determine the use of toxic substances by those involved in traffic accidents. It must be taken into account that our laboratory only receives samples from people who died after the accidents, and we do not have other data.

## Conclusion

This study presents the prevalence of ethanol and drugs found in traffic accident deaths in Galicia in a period of 10 years. The toxicological findings provide information on consumption habits, helping to increase knowledge about the most consumed substances and about the profiles of the victims. This is of great importance to learn more about this type of death and be able to define education and prevention protocols, evaluating the effectiveness of the legislation or the need for more controls on the road by the authorities.

## Data Availability

The data that support the findings of this study are available on request from the corresponding author.
